# Laparoscopic Surgery for Seminal Vesicle Cysts and Ureterocele with Urination Disorder: A Case Report of Zinner Syndrome

**DOI:** 10.1089/cren.2018.0008

**Published:** 2018-03-01

**Authors:** Takeshi Maehana, Fumimasa Fukuta, Ko Kobayashi, Megumi Hirobe, Toshiaki Tanaka, Naoya Masumori

**Affiliations:** Department of Urology, Sapporo Medical University School of Medicine, Sapporo, Japan.

**Keywords:** seminal vesicle cyst, ureterocele, Zinner syndrome, laparoscopic surgery

## Abstract

***Background:*** Zinner syndrome is defined as seminal vesicle cysts with ipsilateral renal agenesis and an ectopic ureter. Symptomatic cases are very rare. In this article, we present a laparoscopic approach for a case of Zinner syndrome.

***Case Presentation:*** The patient was a 21-year-old male with difficult urination. A right seminal vesicle cyst and right kidney agenesis associated with ureterocele were found on examination and he was diagnosed with Zinner syndrome. First, we performed transperineal puncture of the ureterocele because it closed the bladder neck during voiding. Although voiding symptoms temporarily improved, the ureterocele recurred soon and the urination disorder was reexacerbated. Next, we selected laparoscopic removal of the ureterocele and the seminal vesicle cyst. The procedure was performed with transperitoneal access using four trocars. Perioperative and postoperative complications were not observed, and micturition was satisfactory after surgery.

***Conclusion:*** Treatment for difficult urination because of Zinner syndrome is mostly selected puncture of the ureterocele or seminal vesicle cyst. However, some patients experience recurrence. The laparoscopic approach is minimally invasive and provides a satisfactory surgical field. Therefore, it should be considered the method of treatment for symptomatic Zinner syndrome.

## Introduction

Renal agenesis associated with ipsilateral seminal vesicle cysts, an ectopic ureter opening into the seminal vesicle, and obstruction of the ejaculatory duct is a Wolffian duct anomaly known as the Zinner syndrome.^1^ Iwasaki et al.^2^ regarded this syndrome as a manifestation of an ectopic ureterocele. To date, slightly >200 cases of Zinner syndrome have been described in the literature based on imaging and pathologic examinations. Although this syndrome is generally asymptomatic, it may cause symptoms such as perineal pain, painful ejaculation, hematospermia, abnormal micturition, lower urinary tract symptoms, and dysuria.^3^ If there are any symptoms, the treatment options are surgical resection, puncture, aspiration, and transurethral resection. In this article, we present the first case of a Japanese patient who was managed successfully through a laparoscopic removal of a large seminal vesicle cyst and ipsilateral renal agenesis with ureterocele.

## Presentation of Case

**FIG. 1.**
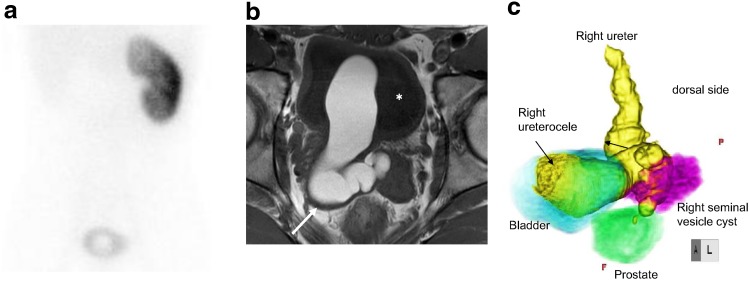
**(a)** 99mTc-dimercaptosuccinic acid scintigraphy showed a defect behind the bladder and right renal agenesis. **(b)** T1-weighted magnetic resonance image demonstrating tubulocystic lesions (*arrows*) behind the bladder (*asterisk*). The high T1 signal intensity suggests proteinaceous content. **(c)** Three-dimensional magnetic resonance image.

**FIG. 2.**
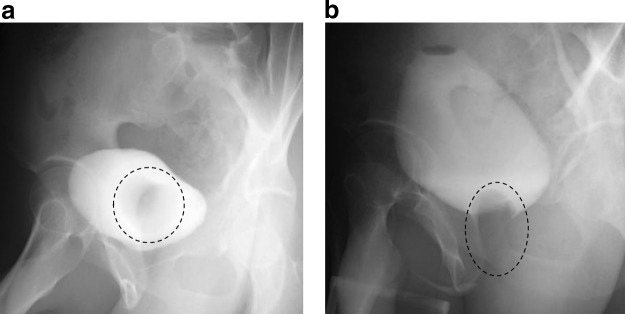
Voiding cystourethrography. **(a)** Before initiation of micturition. **(b)** When urinating. The *dotted line* indicates the ureterocele.

A 21-year-old male had a urination disorder and visited another urologic clinic. He had been aware of difficult voiding and a decrease of voided volume since he was 15 years old, and it gradually worsened. He started treatment with a diagnosis of chronic prostatitis, but his symptoms were not improved. Cystoscopy was performed and a right large ureterocele without a ureteral orifice was found. Then he was referred to our hospital for further evaluation and treatment for the right ureterocele. On uroflowmetry, the maximum flow rate (Qmax) was 3.1 mL/sec. Contrast-enhanced abdomen computed tomography and 99mTc-dimercaptosuccinic acid scintigraphy showed a right ureterocele and right renal agenesis (Fig. 1a). Magnetic resonance imaging demonstrated tubulocystic retrovesical structures with T1 and T2 hyperintense content, and a fluid level suggesting proteinaceous or hematic content (Fig. 1b, c). In voiding cystourethrography, the ureterocele fell into the bladder neck at the time of voiding, which was thought to be the cause of the difficult urination (Fig. 2).

**FIG. 3.**
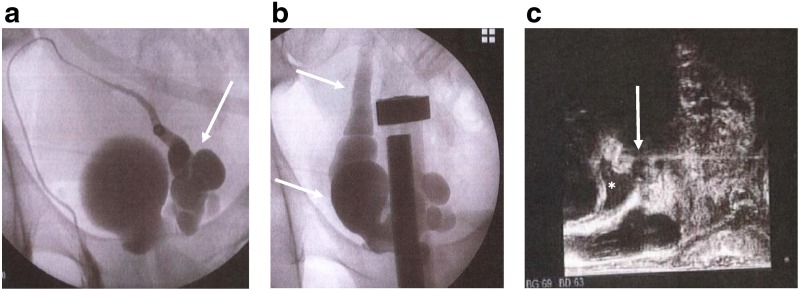
**(a)** and **(b)** Right vasoseminal vesiculography. **(a)** Multiple enlarged seminal vesicle cysts (*arrow*) are enhanced like beads from near the vas deferens. **(b)** A ureterocele and the right ureter (*arrows*) are contrasted. **(c)** Transcranial needle puncture of the ureterocele under transrectal ultrasound guidance. The *asterisk* indicates the ureterocele and the *arrow* the needle.

To confirm the diagnosis and decide the treatment, we performed right vasoseminal vesiculography and transperineal puncture of the ureterocele under anesthesia. In vasoseminal vesiculography, multiple enlarged seminal vesicle cysts were imaged from near the ampulla of the ductus vas deferens; then a ureterocele and the right ureter were visualized subsequently (Fig. 3a, b). The ejaculatory duct was not drawn out. Based on all the examinations, we diagnosed Zinner syndrome. We drained 40 mL of fluid from the ureterocele by transperineal puncture (Fig. 3c) and confirmed by cystoscopy that the ureterocele protruding into the bladder had shrunk. On uroflowmetry after the puncture, Qmax was 20.3 mL/sec and his subjective symptoms were improved. However, slow stream recurred 1 month after the puncture, and Qmax had fallen to 3.7 mL/sec on uroflowmetry. Therefore, we thought that this patient was indicated for laparoscopic removal of the seminal vesicle cyst, the ipsilateral remnant of the kidney if it existed, and the ureterocele.

### Surgery

**FIG. 4.**
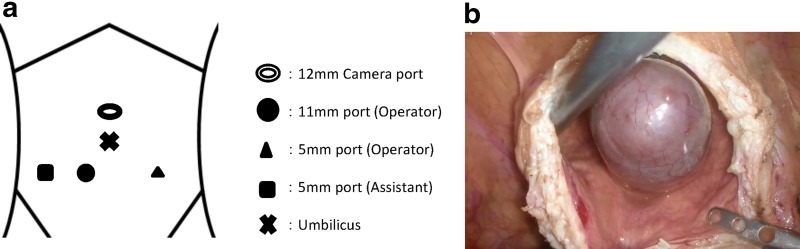
**(a)** Trocar placement. **(b)** View after incision of the bladder and protruding ureterocele.

The procedure was performed through transperitoneal access. The patient was placed in the Trendelenburg (25 degrees) and lithotomy position with four ports. A camera port was added 3 cm above the umbilicus for 0-degree optics. The other ports were located below the umbilicus in a straight line (Fig. 4a). First, we detected the dilated right ureter in the retroperitoneal space and retrogradely dissected it above the iliac vessels. There was tissue suspected of being hypoplastic kidney in the top of the ureter. The lower ureter was dissected toward the bladder. Although the intramural ureter and ureterocele were peeled as much as possible, we gave up approaching the bladder from outside because the ureterocele prominently protruded into the bladder. Then, the right vas deferens was identified and dissected. The dissected vas deferens was peeled off toward the prostate and multiple enlarged seminal vesicle cysts were identified. We peeled seminal vesicles from surrounding tissues to the degree possible. Next, the bladder wall was dissected and a massive ureterocele was identified (Fig. 4b). The ureterocele was very close to the left ureteral orifice and internal urethral orifice. The ureterocele was incised and the turbid fluid content discharged. We observed the ureterocele lumen and confirmed that the ureterocele was in communication with seminal vesicle cysts. Therefore, we diagnosed it as an ectopic ureter entering into a cystic seminal vesicle. The ureterocele was dissected so as to peel it from the bladder muscle layer, and the right ureter, seminal vesicle cyst, and right vas deferens were extracted en bloc from the dorsal side of the prostate. The specimen was placed in an Endopouch Retreiver^®^ (Ethicon, USA) and removed through the camera port. The defect of the bladder was made watertight in two layers using 3-0 Vicryl^®^ (Ethicon). One drain was left at the end of the operation. The operative time was 289 minutes and blood loss was 20 mL. No complications were observed.

### Outcome

Two weeks after the operation, voiding cystography was performed and the urethral catheter was removed. After that, he voided without trouble and 1 year after the operation, his voiding condition was entirely favorable. Uroflowmetry showed that Qmax was 15.8 mL/sec. There were no difficulties in erection and ejaculation.

## Discussion

Zinner syndrome, which is considered a male counterpart of the Mayer-Rokitansky-Küster-Hauser (MRKH) syndrome seen in females, was first described by Zinner in 1914.^1^ It is a rare congenital urological anomaly of the mesonephric duct. This syndrome is usually asymptomatic. However, a ureterocele or a seminal vesicle cyst can grow and induce inflammation and stimulate the surrounding viscera, resulting in bladder-irritating symptoms, including urinary frequency, dysuria, suprapubic pain, hematospermia, and painful ejaculation. Treatment of this syndrome can be decided according to the existing symptoms. If the symptoms are mild, percutaneous drainage, or transrectal or transurethral aspiration of the ureterocele or seminal vesicle cyst is suitable. However, this approach was reported to have poor therapeutic effects with a high probability of recurrence and the exposure of a young patient to the possible need to repeat the procedure.^3^ Our case also recurred at the first month after puncture. Therefore, for severely symptomatic patients, invasive treatments should be suggested. Various invasive techniques have been proposed for the operative management of seminal vesicle diseases. Historically, open surgery performed through a transvesical, retrovesical, transperineal, or transcoccygeal route has been considered to be the definitive treatment option because of the high success rate reported, but the related morbidity is also considerable. Transurethral resection with unroofing has been reserved for smaller lesions in close contact with the prostate, and is associated with a significant failure rate. Recently, laparoscopic surgery appears to be the most suitable surgical treatment. Carmignani et al.^4^ described the first laparoscopic excision of a seminal vesicle cyst associated with ipsilateral renal agenesis and about 20 cases of laparoscopic excision have been reported to date. Laparoscopic surgery has the advantages of direct access to the seminal vesicle with excellent images in the deep and retrovesical fields, with minimal invasiveness compared to open resection. There have been many reports of using a four-port transperitoneal approach as in our case. Jang et al.^3^ reported the successful application of laparoendoscopic single-site surgery. However, advanced skills are often needed for laparoscopic surgery even if it is not single-site surgery because some of the procedures require ureteral reimplantation or bladder reconstruction as in our case. Recently robot-assisted laparoscopic management has been reported to play a primary role in the treatment of this condition. However, we cannot use a robot for this anomaly because the Japanese health insurance system restricts the diseases for which we are permitted to use this technique.

## Conclusion

Seminal vesicle cysts and right kidney agenesis associated with a ureterocele are rare, but they should be considered in men with otherwise inexplicable voiding symptoms, perineal discomfort, or other genitourinary complaints of unclear etiology. Laparoscopy is considered to be minimally invasive for management of these combined anomalies, providing excellent intraoperative access and visualization without postoperative morbidity.

## Abbreviation Used

Qmax: maximum flow rates
